# Leveraging FMMEA for Digital Twin Development: A Case Study on Intelligent Completion in Oil and Gas

**DOI:** 10.3390/s25185846

**Published:** 2025-09-19

**Authors:** Nelson Victor Costa da Silva, Flavia Albuquerque Pontes, Mariana Santos da Silva, Breno Cagide Fialho, Jamile Eleutério Delesposte, Dalton Garcia Borges de Souza, Luiz Antônio de Oliveira Chaves, Rodolfo Cardoso

**Affiliations:** 1 Instituto de Ciência e Tecnologia (ICT), Universidade Federal Fluminense (UFF), Rio das Ostras 28890-000, Brazil; nelsonvictor@id.uff.br (N.V.C.d.S.); fpontes@id.uff.br (F.A.P.); brenofialho@id.uff.br (B.C.F.); daltonborges@id.uff.br (D.G.B.d.S.); luizchaves@id.uff.br (L.A.d.O.C.); rodolfo_cardoso@id.uff.br (R.C.); 2 Escola de Engenharia de Petrópolis (PEP), Universidade Federal Fluminense (UFF), Petrópolis 25650-050, Brazil; jamile_delesposte@id.uff.br

**Keywords:** failure, Digital Twin, maintenance, oil and gas, completion system, decision support systems

## Abstract

The implementation of Digital Twins (DTs) represents a significant advancement for the Oil and Gas (O&G) industry. A DT virtually replicates a physical asset, enabling the monitoring, diagnosis, prediction, and optimization of its outcomes. Since failures are undesirable outcomes, investigations into potential failure modes are often integrated into the development. Traditional methods, such as Failure Modes and Effects Analysis (FMEA) and Failure Mode, Effects, and Criticality Analysis (FMECA), are widely used to identify, assess, and mitigate risks. However, there is still a lack of specific guidelines for studying potential failures in complex systems. This article introduces a framework for Failure Modes, Mechanisms, and Effects Analysis (FMMEA) as a tool for identifying and assessing failures in early DT development. Exploring failure mechanisms is highlighted as essential for effective prediction and management We also propose adjustments to FMMEA for complex, predictable systems, such as using a DPR (Detectable Priority Risk) instead of RPN (Risk Priority Number) for prioritizing risks. A comprehensive case illustrates the framework’s application in developing a DT for an intelligent completion system in a major O&G company. The approach enables mechanism-oriented failure analysis and more detailed prognostic health management, providing greater transparency in the failure identification process.

## 1. Introduction

For more than a century, the oil and gas (O&G) industry has been vital to the global economy, impacting energy supply through complex exploration, production, and distribution processes. Although fueled by advanced technology, the industry faces growing challenges and pressure to operate more sustainably, safely, and efficiently. In this context, Digital Twins (DTs) have emerged as transformative solutions [1,2,3].

A DT (Digital Twin) is a virtual replica of a real-world object or process, maintained in synchronization through specific intervals and levels of detail, mirroring its physical counterpart by using historical and real-time data to simulate past, present, and future behaviors [4,5]. The FMMEA (Failure Modes, Mechanisms, and Effects Analysis)emerges as a strategic tool for the development of DTs, enabling the systematic identification of failure modes, their causes, and effects, supporting accurate modeling of the behavior of physical assets and the prioritization of critical elements. Ref. [6] provides a definition of DT widely used across industries, stating that a DT is “a set of virtual information constructs that mimics the structure, context, and behavior of a unique physical asset or a group of physical assets, and is dynamically updated with data from its physical twin throughout its lifecycle, informing decisions that realize value.” In the Oil and Gas (O&G) industry, integrating FMMEA into DT development adds value by enhancing operational capabilities, providing detailed insights into production chain processes, and increasing system reliability [7,8]. This approach enables optimized production, smarter asset management, and efficient integration of multiple data sources, resulting in higher efficiency and productivity [9,10]. By integrating AI, IoT, Big Data, and cloud computing, DTs supported by FMMEA advance process optimization and provide deeper insight into industry assets [11,12,13].

DTs are spreading across multiple sectors. In manufacturing, surveys synthesize use cases in virtual commissioning, quality control, predictive maintenance, and reliability analysis through FMMEA integration throughout the product and production lifecycles [14]. In the built environment, systematic reviews report DT adoption for life-cycle monitoring, BIM-integrated asset management, energy optimization, and facility operations, while also mapping barriers and research directions [15,16]. Incorporating FMMEA into DTs enables systematic identification of critical failure modes, improving model fidelity and supporting preventive actions. In the energy domain, comprehensive reviews of smart grids discuss DT-enabled situational awareness, forecasting, and real-time optimization alongside grid-scale data complexity, interoperability, and cybersecurity challenges [17]. FMMEA-driven DTs in this domain allow prioritized risk assessment of key components, guiding maintenance and operational decision-making under uncertainty. In healthcare, applications range from hospital operations to personalized medicine, with DT-FMMEA approaches offering structured reliability assessment and risk mitigation strategies while emphasizing governance, privacy, and validation issues in data-intensive settings [18]. An applied example in smart buildings illustrates DT integration with IoT, FMMEA-informed reliability evaluation, and data-driven control for indoor environment and energy management [19]. Across sectors, recurrent limitations include interoperability and standardization across heterogeneous assets, models, and data spaces; data governance, privacy, and cybersecurity for high-frequency and sensitive telemetry; model fidelity, verification/validation, uncertainty quantification, and lifecycle maintenance of DT models; scalability of streaming data ingestion, edge–cloud orchestration, and cost/skills constraints for enterprise deployment; and limited benchmarking and reproducibility across contexts. FMMEA integration addresses these gaps by providing structured methods for risk assessment, reliability-based model validation, and proactive maintenance strategies, contributing to cross-sector solutions for common reference architectures, robust data–model fusion, and sustainable DT operation at scale.

In the O&G industry, the adoption of DTs is aligned with sector demands to enhance operational performance. These models are developed to optimize process monitoring, control, and management across exploration, drilling, and production [20,21,22,23]. FMMEA-enhanced DTs allow identification and prioritization of critical failure modes in complex subsystems, improving reliability, reducing downtime, and guiding preventive and corrective actions. They also target resource allocation and production outcomes [24,25,26] and underpin predictive analytics for operational decision-making [27,28,29,30,31,32]. Within Prognostics and Health Management (PHM), DTs support continuous condition monitoring, early anomaly and failure detection, and maintenance forecasting, enabling proactive actions to preserve system integrity [28,29,30,31]. When combined with FMMEA, hybrid analyses not only fuse sensor data with physics-based or simulation models for real-time state estimation but also systematically identify potential failure modes, assess their criticality, and guide preventive maintenance and anomaly detection [29]. Applications extend to integrity management and safety-critical use cases, including well and pipeline leak detection [33,34], with continuous DT-FMMEA-based supervision used to sustain availability, mitigate risks, and extend asset life [32].

Detection and classification of undesirable events are increasingly relevant tasks in various human-monitored activities [35]. In the context of Prognostics and Health Management (PHM) for the O&G industry and in the development of DTs, failure identification is a fundamental step before predictive analytics and anomaly detection. It provides insights into failure modes, mechanisms, and causes, facilitating the identification of patterns or signs that may indicate future problems. However, the effectiveness of this identification depends on several factors, such as data quality and availability, the complexity of the systems analyzed, and the techniques and methods applied [36].

The implementation of DTs in the energy sector is strongly supported by methodologies such as Failure Modes and Effects Analysis (FMEA) and Failure Mode, Effects, and Criticality Analysis (FMECA). FMEA, which is widely applied [37,38,39], offers a systematic approach for identifying potential failure modes and their effects on operations, enabling preventive actions. FMECA goes further by including a criticality analysis, providing a deeper understanding of the impact of failures [40].

Several authors have proposed extensions to these methods [41,42,43], such as the Dynamic Hybrid FMECA, which combines techniques like the Entropy Method and the Best-Worst Method. These innovations enhance reliability analysis by considering dynamic interactions and complex failure mechanisms, making them especially suitable for the complexities involved in DTs [44].

Beyond traditional methodologies such as FMEA and FMECA, the O&G industry employs other analytical tools to identify potential failures. Fault Tree Analysis (FTA) is widely used, adopting a top-down deductive approach to analyze undesired system states, using Boolean logic to connect various lower-level events. This technique is particularly useful for understanding complex systems and has been enhanced with fuzzy logic to deal with data uncertainties, resulting in more accurate failure probabilities [45,46].

Similarly, the Hazard and Operability Study (HAZOP) provides a structured, systematic analysis of processes, focusing on the identification and management of risks. Its application in oil production units has been documented for risk identification and the generation of mitigation recommendations [47]. Event Tree Analysis (ETA) complements these approaches by providing a forward-looking analysis that maps possible outcomes from an initiating event. ETA is often used together with FTA to enhance safety barrier reliability assessments [48].

Although traditional methods like FMEA, FMECA, and FTA offer risk identification and management, they often fail to address the dynamic and complex interactions characteristic of these systems and are not well-suited for assessing the propagation of failure mechanisms—an important predictive capability for DTs. Failure Modes, Mechanisms, and Effects Analysis (FMMEA) goes beyond these limitations by incorporating a detailed exploration of failure mechanisms, which are important for effective prediction and management of potential failures in DT systems. According to ISO [49], a failure mechanism is a process leading to failure, which may be physical, chemical, logical, or a combination of these. FMMEA is designed to dissect and understand the intricate mechanisms that can lead to failures, making it particularly effective for DTs, which require a thorough analysis to accurately replicate physical assets. This approach not only improves DT reliability but also enhances their effectiveness in predictive maintenance strategies. By focusing on mechanisms, FMMEA provides insights for optimizing maintenance schedules and preventive actions that traditional methods might overlook.

Although several studies have investigated failure identification in various industries using different methodologies [50,51], their adoption in the oil, gas, and energy industries is still relatively limited [52,53,54]. The O&G sector stands out from others due to its significant environmental impact, high capital requirements, specific regulatory challenges, a global supply chain, and, above all, high-risk operations [55]. Furthermore, there is a lack of comprehensive guidelines for the effective implementation of failure identification methods, especially in the context of DT development. This gap in the literature offers an important opportunity for the development of tailored frameworks that address the unique challenges and complexities of O&G systems, improving the reliability and predictive capabilities of DTs.

This research aims to fill this gap by developing a comprehensive framework for the implementation of FMMEA in the O&G industry, with special application to DT development. Recognizing the limitations of traditional methods such as FMEA, FMECA, and FTA in addressing the dynamic and complex interactions typical of DT environments, our study focuses on improving the reliability and predictive capabilities of DTs through a detailed, mechanism-centered analytical approach [50,56,57]. By introducing an FMMEA implementation framework tailored to the unique demands of DT systems, we aim to provide practical guidelines that facilitate the identification and mitigation of potential failures. To demonstrate the practical application of FMMEA, we also present a case involving an Inflow Control Valve (ICV), which is part of an intelligent completion system (ICS)—an Industry 4.0-oriented approach used by one of the world’s leading O&G companies. To highlight and demonstrate the advantages of FMMEA, Table 1 presents a brief comparison between the aforementioned methodologies, highlighting their purpose and advantages. Unlike other approaches, FMMEA provides a mechanism-based analysis that reveals distinct effects and insights, offering predictive capabilities that many traditional methods may lack. Thus, FMMEA shows a significant impact on studies and simulations of events resulting from possible functional failures, which can be aligned with predictive tests necessary for the development of the Digital Twin.

Thus, FMMEA shows a significant impact on studies and simulations of events resulting from possible functional failures, which can be aligned with predictive tests necessary for the development of the Digital Twin.

**Figure 1:**
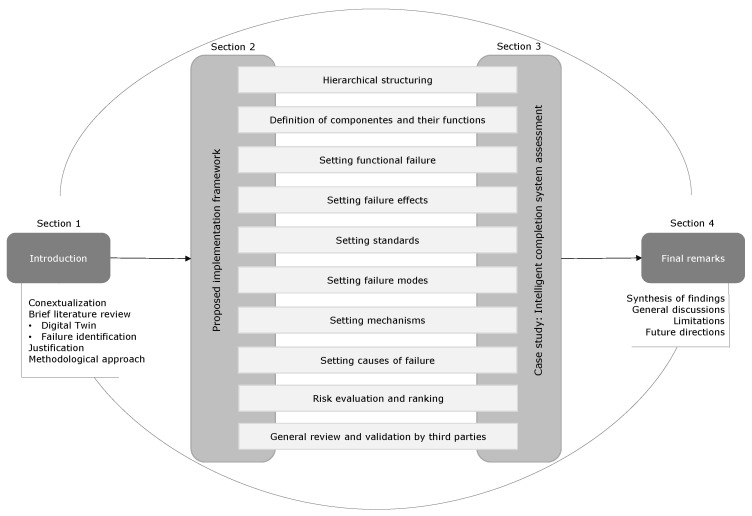
Article’s methodological map.

The remainder of this paper is organized into three sections. Section 2 presents a detailed framework for implementing failure identification, outlining step-by-step procedures tailored for applications in the O&G. Section 3 applies the FMMEA framework to a real-world scenario, demonstrating its practical benefits and effectiveness in identifying and mitigating potential failures. Finally, Section 4 synthesizes the findings, discusses the implications for industry practice, and suggests directions for future research, highlighting the value of the proposed FMMEA framework in advancing risk assessment methodologies in O&G. Figure 1 summarizes the structure of the article in a methodological map, illustrating how the sections are interconnected.

**Table 1:** FMECA, FMEA, FMMEA and FTA comparison [58,59,60,61].

Methodology	Objective	Advantages of Use
FMEA	Identify and prioritize potential failures in equipment, systems or processes [58].	It allows the creation of a hierarchy of potential failures and provides recommendations for actions aimed at avoiding them through maintenance techniques [59].
FMMEA	Unite the systematic vision of FMEA with the idea of “design for reliability”, in order to identify high-priority failure mechanisms, and the knowledge about causes and mechanisms obtained by FMMEA [60].	It assists in product development, including virtual qualification, accelerated testing, root cause analysis, and lifetime consumption monitoring. “All the technological and economic benefits provided by these practices are best realized through the adoption of FMMEA” [60].
FMECA	Allow the classification of critical failure modes and effects taking into account their probabilities of occurrence and the severity of their effects [59].	It strengthens the criticality analysis with a quantitative character [59].
FTA	Represent in a graphical and standardized way the failures of a system and their respective effects or top events, unfolding them in a logical tree up to the root causes [61].	It allows the creation of a relationship between priority failures and intermediate failures or events up to the root cause in a simple and visual way, with the help of logical symbols to assist the reader [61].

## 2. Proposed Implementation Framework of FMMEA

**Figure 2:**
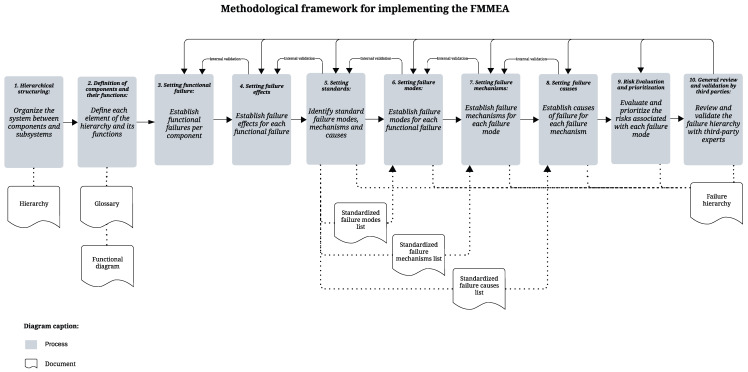
Methodological framework for implementing the FMMEA.

This section presents a guiding framework for the FMMEA (Failure Mode, Mechanism, and Effects Analysis) in the O&G. It addresses two questions: “What are the steps for implementing FMMEA?” and “Which references or approaches can be used at each stage?” The framework comprises ten main steps: hierarchical structuring, defining components and their functions, identifying functional failures and effects, establishing standards, determining failure modes, mechanisms, and causes, evaluating and ranking risks, and conducting a general review with third-party validation (see Figure 2).

Some steps propose the construction of documents that will serve as reference for the development of subsequent steps and can be considered as by-products of the framework. Also, some steps incorporate small adjustments to traditional FMMEA, in order to make it more suitable for DT development with predictive capabilities.

To anchor the framework in established practice, we use international standards as a common foundation. In particular, ISO 14224 provides the asset taxonomy and coding conventions we adopted to harmonize entries for modes, mechanisms, and causes, while the ISO/NBR vocabulary keeps definitions consistent across all steps [49,62]. Rather than replicating prescriptive procedures, the framework maps each activity to these standard concepts and data fields, enabling interoperability with reliability/maintenance databases and the traceable exchange of results. Where project-specific rating scales are required (e.g., severity, frequency, detectability), we maintain semantics consistent with industry guidance and document the mapping used in risk prioritization.

Although the steps of the framework can be applied to various industrial contexts, in this study they were specifically structured to support the development of DTs in the oil and gas sector. The focus on O&G stems from the criticality of its assets, which operate under severe conditions and involve high costs and risks in case of failure. In such environments, ensuring systematic and standardized identification of failure modes and mechanisms is essential to enable predictive capabilities. By integrating FMMEA outputs into DTs, the framework provides not only a comprehensive mapping of potential failure scenarios but also a structured data flow that feeds predictive models with reliable information. This alignment facilitates the continuous calibration of DTs, helping to preserve model accuracy over time and ensuring that risk assessments remain consistent with real operational conditions.

The framework also includes intermediate checks, such as internal validations, which take place between each stage from the third to the eighth step. These validations are intended to ensure coherence, deliberate on additional factors, improve analyses, and guarantee comprehensive recognition of potential scenarios within the identifications and connections established throughout the implementation of the framework.

**Figure 3:**
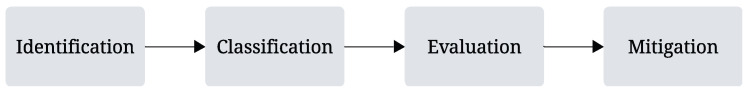
Typical FMMEA/FMEA structure.

In Figure 3, the structure of an FMMEA, or even a traditional FMEA, is presented, comprising the phases of identification, classification, evaluation, and mitigation. In the identification phase, the focus is on recognizing and listing: system components, their expected functions, potential functional failures, failure modes (ways in which the failure can occur), failure mechanisms (physical or chemical processes leading to failure), and failure causes. This is a broad phase of technical mapping aimed at understanding the system and its potential points of vulnerability. In the proposed framework, Figure 2, this phase corresponds to activities 1 through 8, with the necessary specifications for the case under analysis [63].

Once the elements and potential failures are identified, the process moves on to the classification phase, in which each failure is evaluated based on criteria such as: Severity of effects (impact of the failure), Probability of occurrence, and Detectability. This classification usually follows standardized scales (e.g., from 1 to 10) and results in risk indices such as the RPN (Risk Priority Number).

In the evaluation phase, a critical analysis of the identified risks is conducted, asking: Which failures are most critical? Which risks pose the greatest threat to performance, safety, or operational continuity? Which require prioritized attention? This is the moment to prioritize actions and deepen the analysis of the most relevant failure modes. In the proposed framework, the classification and evaluation phases are covered in activity 9 (Figure 2).

The final Mitigation phase may propose corrective, preventive, or control actions, such as design modifications, material improvements, adoption of sensors for monitoring, implementation of safety barriers, or predictive maintenance plans. The objective here is to reduce the probability or impact of failures—or to detect them in advance. In the case of this FMMEA study applied to DTs, mitigation will be carried out through the monitoring and control of sensor-equipped components and critical failures identified in this FMMEA. It may also incorporate predictive models to estimate the future behavior of failures. Therefore, the detailed development of how mitigation will be implemented is not the focus of this study.

The choice of FMMEA as the central method for risk assessment, rather than purely data-driven approaches, was based on a critical analysis of the characteristics, advantages, and limitations of each approach, especially in the context of complex applications such as DT systems in the oil and gas industry.

Data-driven approaches, including machine learning techniques, data-driven reliability models, and statistical analysis of failure histories, offer considerable advantages in terms of automation, rapid pattern detection, and predictive capability in contexts where there is a large availability of high-quality data, which was precisely the case in the study at hand. However, their applicability may be limited in complex, emerging, or innovative systems where historical data is scarce, incomplete, or not yet available. Furthermore, although these approaches are effective at identifying correlations, they do not always provide a causal understanding of the underlying failure mechanisms [64].

In this regard, FMMEA stands out by adopting a structured and expert-driven approach. The main advantage of FMMEA lies precisely in its ability to model the system’s technical and operational knowledge even in the absence of large volumes of data, providing a robust and transparent analysis to support design, maintenance, and monitoring decisions [65].

Moreover, FMMEA allows for the incorporation of multiple knowledge sources (technical manuals, engineering experience, regulatory standards, computational simulations, etc.), which is essential for applications such as DTs, where it is necessary to represent not only past behavior but also expected performance under varying operational conditions [63].

Another relevant factor is the explainability and traceability of FMMEA. Unlike some machine learning algorithms that operate as “black boxes,” FMMEA offers a clear logical structure for risk prioritization and the definition of mitigation actions.

### 2.1. Hierarchical Structuring

**Figure 4:**
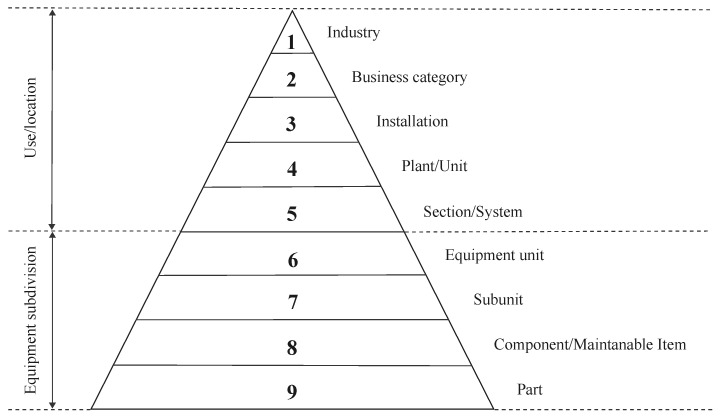
Taxonomy classification with taxonomic levels [49].

The initial step involves listing the various components that comprise the equipment, organizing them into a hierarchical structure based on common characteristics such as physical location or operational functionality. Classification begins at the system level and continues to the smallest relevant level, typically components subject to maintenance. In addition, this stage includes the assignment of codes to the different elements based on their position within the hierarchy. An example of hierarchical classification can be seen in Figure 4.

### 2.2. Definition of Components and Their Functions

The second step involves describing the components and their functions, resulting in the creation of a document that consolidates the definitions of all components identified in the previous step. At this stage, primary and, when relevant, secondary functions are defined for each component. Ref. [63] highlights the importance of defining functions using an active verb and a noun, focusing on the main function of the system, subsystem, or component under analysis. This information can be obtained through a literature review, manufacturer documentation, consultations with experts, and interactions with equipment suppliers. In addition to the definitions and functions, it is also valuable to include representative or actual images of these items, as such images aid in system understanding and analysis. The document that compiles the definitions and functions of equipment is referred to as the taxonomic glossary. This document is organized according to the hierarchy established in the previous step.

Clearly defining each item’s functions is essential for identifying failures, which usually result from unmet functional requirements. It also facilitates defining interfaces between components and subsystems [66].

### 2.3. Setting Functional Failure

Ref. [67] defines functional failure as the condition in which a component is unable to perform one of its functions as expected by users. This includes not only the inability to fulfill its primary function but also failures in secondary or additional functions that the component may have. In other words, a functional failure occurs when any specific function of a component does not meet the desired performance standards.

Thus, for the third step of the framework, based on the primary functions established in the previous step and represented in the functional diagram, the analysis of all possible functional failures of the equipment is initiated. As described by ISO [49], four conceptual points can be considered: the type of global system under analysis, the location of each component within that system, the main category of the equipment, and its primary function within its subsystem. The Functional Diagram is used to structure information about each hierarchical component, helping the team move forward with this step.

### 2.4. Setting Failure Effects

The fourth step of the framework concerns establishing the failure effects, defined as the anticipated consequences that a failure may have on the system [68]. The association of failure effects is performed through the analysis of the possible adverse outcomes resulting from functional failure. Each failure effect–functional failure link examines component function, potential adverse scenarios, and subsystem impacts.

### 2.5. Setting Standards

The fifth step starts with the establishment of a standardized language for mode, mechanism, and cause, in order to provide greater robustness and reliability to the FMMEA framework. In the oil, petrochemical, and natural gas industries, there is great attention paid to equipment safety, availability, reliability, and maintainability. In this context, the treatment and analysis of data on failures, mechanisms, and causes of failure in these facilities and operations have become even more relevant. Therefore, it is necessary to establish a standardized language that unifies the information to be shared among various parties and disciplines, both within the same company and between different companies [49].

Furthermore, if there is no standardized language available, it is advisable to use the standards adopted by ISO [49]. As an international standard, ISO 14224 provides coding rules already widely used in the petrochemical market, with information focused on maintenance, which can facilitate communication among all parties involved in the framework process.

### 2.6. Setting Failure Modes

ISO [49] failure modes are understood as the manner in which failure occurs. Refs. [69,70] state that the mode defines how a failure is physically observed, whether through visual inspection, electrical measurement, or other tests and measurements. The potential mode can be identified by understanding how its deficiency may manifest itself [69]. Ref. [71] affirms that failure modes are closely related to the product’s functional and performance requirements.

To identify possible and unlikely failure modes, it is important to understand the component’s life cycle and the environmental and operational loads that occur during the manufacturing, transportation, operation, and storage phases. These loads can be obtained from manuals, standards, and previous experience [50,69].

As previously mentioned, failure modes in FMMEA are standardized for the O&G industry by adopting established standards or models like ISO 14224:2016. Once standards have been defined, possible failure modes for each component–functional failure relationship are identified. When defining the most appropriate modes for each functional failure, it is necessary to frequently consult this standardized list. A detailed analysis should be performed for each functional failure and component, verifying whether each listed mode could be related, i.e., whether the mode could demonstrate how the functional failure occurred. Therefore, it is desirable to identify not only failure modes that have already occurred, but also probable and even less likely ones.

Internal validations occur during and/or after each step, especially in steps 6, 7, and 8. These validations aim to verify consistency, consider additional aspects, complement analyses, and ensure that all possibilities are identified in the associations made during framework implementation. A common occurrence is the initial identification of modes, mechanisms, and causes that do not adhere to the analyzed sequence (component, functional failure, and other FMMEA items). These validations help prevent such inconsistencies from persisting.

During internal validations, modifications to previously established steps are common. This is because the validation process involves reviewing all the previously established relationships between component, functional failure, their modes, mechanisms, and even the causes and effects. This validation is carried out by members of the team responsible for developing the FMMEA. Although recommended, internal validation is optional.

It is worth highlighting that each relationship between component, functional failure, and mode opens a new branch in the analysis hierarchy. In other words, modes define a new level in the failure hierarchy.

### 2.7. Setting Failure Mechanisms

A failure mode may be the result of one or more different failure mechanisms [50,72]. Accordingly ISO [49] and NBR [62], the failure mechanism is the process that leads to failure, and it can be physical, chemical, logical, or a combination of these. Ref. [73] mention that the failure mechanism should be identified based on prior experience, similar products, and a comprehensive consideration of possible failure modes.

Just as failure modes can be standardized, failure mechanisms can also be standardized, for example using ISO [49], which provides a reference list of existing possibilities. It is recommended to conduct an analysis for each component, functional failure, and mode relationship to identify mechanisms that could explain the process leading to failure. The mechanism considers not only the mode and its respective component, but also the functional failure. Thus, for the same mode and component, different mechanisms may occur for different functional failures. While this may seem obvious, it is often overlooked in analyses involving numerous components and modes. It is essential to list all possible mechanisms, including the less likely ones. Internal validations should also be performed during or after the definition of mechanisms.

Failure mechanisms are critical in evaluating the role that the environment plays in accelerating failure. This allows modeling the interactions between life cycle environmental loads and the time to failure (TTF).

The knowledge about mechanisms, combined with the causes obtained through FMMEA, supports efficient product development, including virtual qualification, accelerated testing, root cause analysis, and life consumption monitoring [50].

FMMEA outcomes identify monitoring parameters and relevant Physics-of-Failure models for predicting component lifespan [74].

### 2.8. Setting Failure Causes

Failure causes should be associated with each established failure mechanism. The failure causes defined at this stage are derived from the standardized list of causes defined in step 5. ISO [49], failure causes are defined as the circumstances during design, manufacturing, or use that led to the failure.

Possible failure causes are identified by investigating the conditions throughout the entire product life cycle, including manufacturing, testing, storage, transportation, handling, operation, and maintenance processes [69]. This step is essential to identify one or more root causes of the analyzed failure, pointing the way toward preventive and/or corrective actions. Internal validation should occur through a critical analysis of the selection and association of mechanisms and causes.

Once the failure causes have been established, a first version of the complete failure hierarchy is generated. The failure hierarchy is a systematic approach employed to find the root cause of a functional failure in equipment or a production system. It can be represented by a tree diagram, a spreadsheet, or a virtual dashboard, for example, with the choice made according to team needs.

### 2.9. Risk Evaluation and Prioritization

Ref. [59] emphasize that failures identified through analysis methodologies such as FMMEA or FMEA require prioritization methods for the application of preventive or predictive actions. Based on the analysis of the degrees of severity, frequency, and detection of a given failure, its prioritization becomes necessary, which can be achieved by multiplying each index, resulting in the indicator known as the Risk Priority Number (RPN).

Ref. [75] propose that, to align priority analyses with the specific objectives of a project, it is necessary to adjust the parameters used in the calculation of the RPN. In this context, the Detectability Priority Risk (DPR) was developed, an indicator designed to prioritize failure sequences with greater detection and monitoring capability. The key difference between the DPR and the traditional RPN lies in the ordering of the detection category: in the RPN, the detection category is classified in descending order, where “very high detection” receives a value of 1 and “very low detection” receives a value of 5, and in the DPR, the detection category is classified in the opposite way—from “very low” (1) to “very high” (5). This adjustment reinforces the relevance of failure sequences with higher detection capability.

This change was made to meet the need for building an FMMEA for a DT, as high levels of detectability are essential for DT application, even though low detection capability is more prone to risk.

Therefore, the purpose of the ninth step of the framework is to determine the order of priority for intervention in the failures identified in the FMMEA by means of an indicator, either the RPN or the DPR. Both indicators are calculated by multiplying the severity, frequency, and detection indexes: severity assesses the degree of impact of the failure effect and measures the level of severity of the failure mode; frequency assesses the likelihood of occurrence of the failure mode; and detection assesses the probability of the failure mechanism being detected.

Thus, in the DPR, prioritization is carried out according to the degree of detectability of the failure, that is, the higher the value, the greater the capability to monitor the functional failure. On the other hand, the RPN measures the risk potential in each failure mode, that is, the higher the value, the greater the risk associated with the failure.

### 2.10. General Review and Validation by Third Parties

The final step aims to conduct a comprehensive review and critical evaluation of the complete failure hierarchy, carefully analyzing each functional failure, failure effect, failure mode, failure mechanism, and failure cause established in the previous steps.

The general review should be carried out by the team directly involved in constructing the failure hierarchy, to ensure the coherence of the FMMEA items. This review must be thorough and cover all items in the failure hierarchy to ensure that all aspects are analyzed both individually and in correlation with the others. This activity may include input from the professionals involved and working meetings to implement necessary adjustments.

Subsequently, validation by third parties should be conducted, i.e., by collaborators external to the team that built the hierarchy under analysis, in order to avoid bias in the evaluation. These professionals may be internal or external to the organization, as long as they have not directly contributed to the development of the FMMEA. Validation is key to identify and remove possible inconsistencies in the association of items and to recognize relevant elements that were not associated in previous steps.

Together, the general review and third-party validation ensure that subsequent stages use consistent and accurate data. After review, validation, and adjustments, a new validated version of the complete failure hierarchy is generated. This step closes the framework. The next section provides a real-world example, illustrating the use of this framework in practice.

## 3. Case: Application in an Intelligent Completion System

This section presents a practical application of the FMMEA framework, as introduced in Section 2. This application was carried out for the ICS in the O&G industry, which integrates surface and subsea systems for the remote control of downhole tools, real-time monitoring, and data-driven decision-making.

The well completion system is responsible for preparing and equipping a newly drilled well for production or injection, aiming to maximize its service life and minimize interventions. The intelligent version of this system integrates permanent sensors, transmission cables, and surface-actuated control valves, enabling continuous monitoring, real-time data analysis, and remote operational adjustments. This approach, supported by digital platforms, allows for zonal flow optimization, early detection of issues such as water migration or gas influx, and cost reduction, especially in subsea or hard-to-access environments. By combining technological innovations with traditional practices, intelligent completion enhances operational efficiency and reduces the need for maintenance throughout the well’s life cycle.

The data used to analyze the system’s behavior were obtained from multiple sources, including expert experience, internal technical documentation, technical papers in the field of study, ISO 14224, Technical Specification ET-3000.00-1210-276-PPQ-006, the real failure database of the well observed in the case, and the Offshore Reliability Data Handbook (OREDA). Combining these sources provided practical and normative information that reflects both field experience and internationally recognized standards, ensuring greater consistency and representativeness in the analysis.

In summary, this case study applies the FMMEA framework with the purpose of generating inputs for the construction of a DT of the ICS, enabling the simulation, prediction, and optimization of asset performance based on failure analysis.

The FMMEA was developed as part of a research project within a major O&G company. The main objective of the project was to build a pilot DT model of the ICS for analyzing well control operations, providing support for deviation diagnostics, system integrity analysis, and investigation of symptoms. A team of approximately ten professionals was responsible for developing the FMMEA. In total, 5 subsystems, 25 components, and 35 functional failures were analyzed, resulting in 18 modes, 27 mechanisms, and 11 distinct failure causes, configuring 877 failure sequences.

This section also demonstrates the benefits of the FMMEA framework and the main conclusions regarding the identification and management of potential failures. As an example to illustrate the application of the framework, the ICV was used. The ICV controls the downhole flow in the production tubing [76] and, in many cases, is considered the heart of an ICS.

### 3.1. Hierarchical Structuring

**Figure 5:**
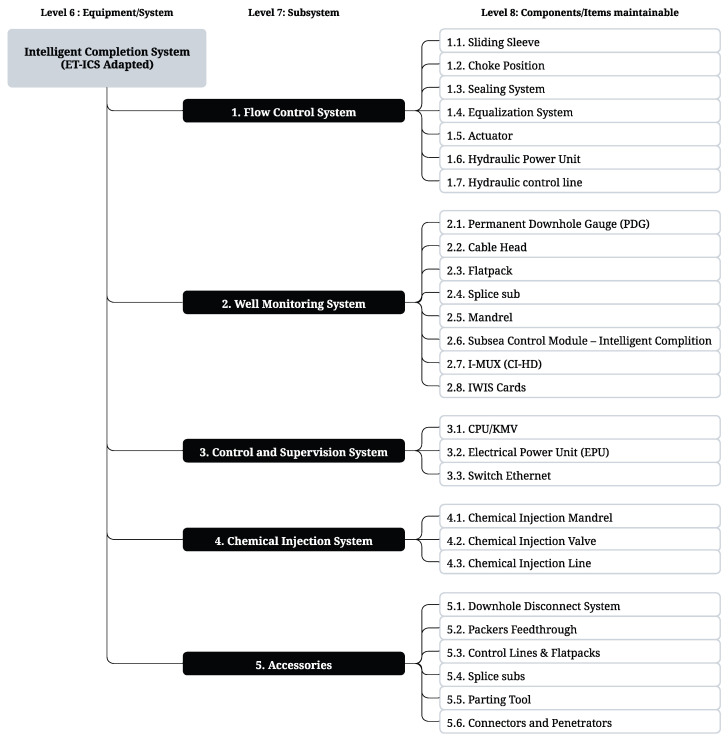
Hierarchical diagram [77].

The framework was applied using a Technical Specification for ICSs to identify the various components constituting the system. Subsequently, components were classified according to the ISO 14224:2006 taxonomy for O&G reliability and maintenance data. The classification process involved allocating the components into one of three hierarchical levels defined by the standard. Specifically, level 6 included a single unit of equipment or system, level 7 encompassed subunits or subsystems, and level 8 included maintainable components or items. The result of this phase was the development of a hierarchical diagram presented as a tree structure, which covered a total of 5 subsystems and 25 individual components. Classification based on hierarchical levels facilitates the comparison of reliability and maintenance data, as well as the assignment of criticality. It also meets the requirement that a failure mode must be attributed to an equipment unit, while a failure mechanism should be attributed to a lower hierarchical level (see Figure 5).

### 3.2. Definition of Components and Their Functions

**Figure 6:**
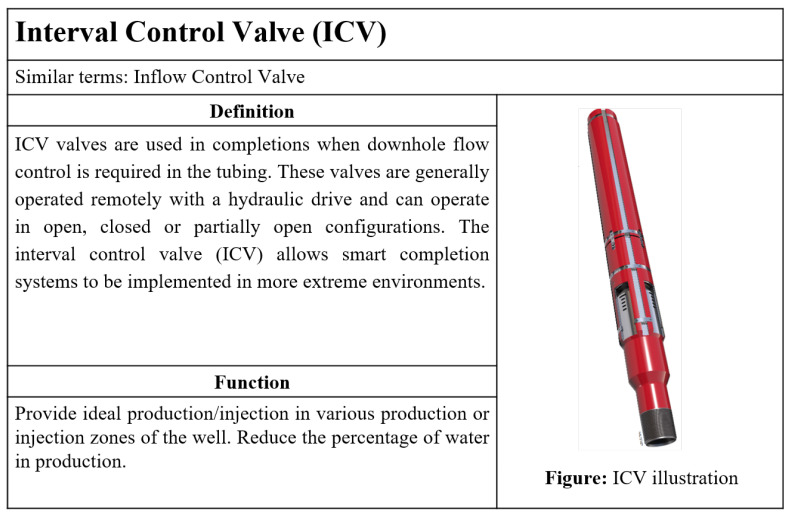
Glossary card—interval control valve.

Based on the literature, each subsystem and component was detailed in dedicated cards. These cards were compiled into an expanded glossary, ensuring each entry included not only a definition but also a collection of synonymous terms from academic sources, the required function, and a visual representation, such as a figure or schematic. For instance, Figure 6 shows this for the ICV.

**Figure 7:**
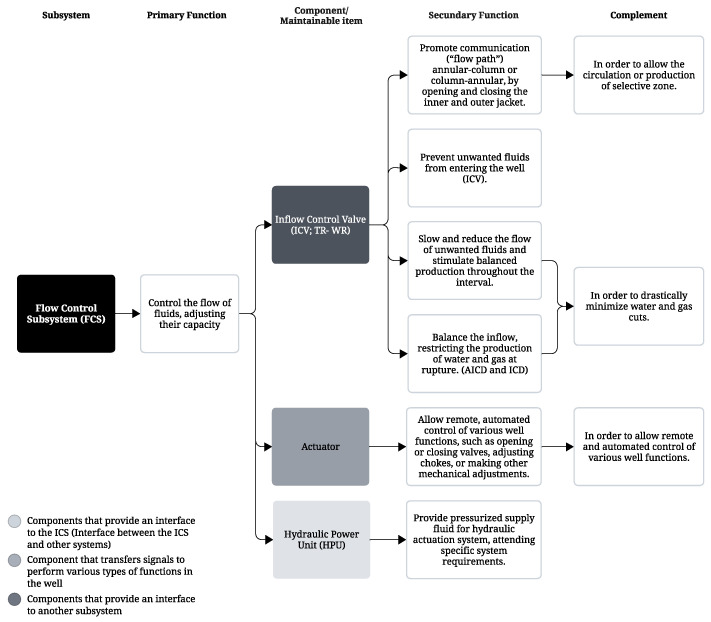
Function tree—flow control subsystem.

Additionally, functional diagrams were developed for all five subsystems to visually represent the connections and interactions between components and their primary and secondary functions. The primary function refers to the role assigned to the subsystem, while the secondary function relates to how the component supports the primary function of its corresponding subsystem, and may be shared by multiple components (see Figure 7).

### 3.3. Setting Functional Failure

In this application, the assignment of functional failures was addressed by analyzing the Functional Diagram and the Taxonomic Glossary of components within the ICS for the wells at the company studied. Figure 7 highlights the flow control subsystem, featuring the ICV, which is the focus of this section.

Definitions and functions from the glossary and internal documents were integrated into the functional diagram, supporting the analysis of parameters affecting remote, hydraulically actuated ICVs. The valve operates with different opening variations, known as choke positions.

With this understanding, the primary function of the ICV in the flow control subsystem was identified: remotely managing pressure and fluid flow in different oil production zones. After clarifying its required function, physical structure, modes of operation, and potential external or internal events that could affect its operation, the following functional failures were assigned:

- 1.3—Failure to actuate the Interval Control Valve (ICV).
- 1.4—Entry of undesirable fluids into the well.
- 1.7—Loss of pressure equalization (fluid flow control in the well).
- 1.8—Valve seizure.

### 3.4. Setting Failure Effects

From the failures mentioned in the previous section, a worst-case scenario analysis was conducted for each effect. The details of each failure effect were briefly described in a sentence that summarizes what the identified adverse scenario represents. Understanding the role of the ICV in the ICS provided insights into the impacts of its failure. When analyzing the scenario of unwanted fluid ingress into production, it implies the entry of water and gas, which reduces the amount of oil produced. In other words, this effect leads to a loss of the system’s financial efficiency.

### 3.5. Setting Standards

To advance the construction of the failure database, the internationally recognized ISO 14224 technical standard was adopted, which is widely used in the O&G industry. This standard enabled the extraction of three tables containing codes related to failure modes, mechanisms, and causes. Specifically, tables B.2 and B.3 of ISO 14224 were used for mechanism and cause codes. Table 2 and Table 3 visually present the first segment of these tables, which were incorporated as secondary pages in the database to facilitate searching and association.

In contrast to the aforementioned data, failure mode codes were categorized by component type, resulting in eight possible categories for components in the O&G sector. For this case study, tables B.7, B.8, B.10, and B.11 from ISO 14224 were used, representing mechanical, electrical, subsea, and completion components, respectively. Since these tables share overlapping codes, they were unified to simplify failure analysis. Table 4 contains the main failure modes addressed by ISO 14224 in table B.11 for well completion.

To reinforce and bring more robustness to the established standard, a document with failure data registered by the companies was also used. This helped develop a comparative reference to the established standard. The data proved consistent with those established by the international standard, validating the use of ISO 14224. Furthermore, to align risk prioritization analyses with project objectives and organizational language, scales and descriptions for severity, frequency, and detectability were adopted from internal technical documentation.

Table 5 and Table 6 show adapted frequency and severity scales based on internal documentation.

**Table 2:** Well completion equipment—failure mechanisms (ISO 14224:2016) [49].

Failure Mechanism		Subdivision of the Failure Mechanism		Description of the Failure Mechanism
Code Number	Notation	Code Number	Notation	
1	Mechanical Failure	1.0	General	A failure related to some mechanical defect but where no further details are known.
		1.1	Leakage	External and internal leakage, either liquids or gases: If the failure mode at equipment unit level is coded as “leakage”, a more causally oriented failure mechanism should be used wherever possible.
		1.2	Vibration	Abnormal vibration: If the failure mode at equipment level is “vibration”, which is a more causally oriented failure mechanism, the failure cause (root cause) should be recorded wherever possible.
		1.3	Clearance/ alignment failure	Failure caused by faulty clearance or alignment.
		1.4	Deformation	Distortion, bending, buckling, denting, yielding, shrinking, blistering, creeping, etc.
		1.5	Looseness	Disconnection, loose items.
		1.6	Sticking	Sticking, seizure, jamming due to reasons other than deformation or clearance/alignment failures.

**Table 3:** Well completion equipment—Failure Causes ISO [49].

Code Number	Notation	Subdivision Code Number	Subdivision of the Failure Cause	Description of the Failure Cause
1	Design-related causes	1.0	General	Inadequate equipment design or configuration (shape, size, technology, configuration, operability, maintainability, etc.) but no further details known
		1.1	Improper capacity	Inadequate dimensioning/capacity
		1.2	Improper material	Improper material selection
2	Fabrication/ installation-related causes	2.0	General	Failure related to fabrication or installation, but no further details known
		2.1	Fabrication failure	Manufacturing or processing failure
		2.2	Installation failure	Installation or assemble failure (assembly after maintenance not included)

Similarly, the detectability table was developed based on the same internal documentation. Table 7 shows the detectability table adapted by the project team, which enabled the development of risk prioritization (RPN) and risk monitoring prioritization (DPR) analyses, both detailed in the following subsections.

**Table 4:** Well completion equipment—failure modes ISO [49].

		Equipment Class Code:	ESP	SS	XD
Failure Mode Code	Description	Examples	Electrical Submersible Pumps	Downhole Safety Valves	Surface Wellhead and X-Mas Trees
AIR	Abnormal instrument reading	False alarm, faulty instrument indication	X		
BRD	Breakdown	Serious damage (seizure, breakage)	X		
CLW	Control-line-to-well communication	Loss of hydraulic control fluids into the wellbore		X	
ELP	External leakage—process medium	Oil, gas, condensate, water; Process medium leak to environment	X		X
ELU	External leakage—utility medium	Lubricant, cooling water, hydraulic fluid, methanol, etc	X		X
ERRO	Erratic output	Oscillating, hunting, instability	X		
FTC	Failure to close on demand	Does not close upon demand signal; Valve(s) fail to close on demand		X	X
FTF	Failure to function on demand	Failure to respond on signal/activation	X		
FTO	Failure to open on demand	Does not open on demand; Valve(s) fail to open on demand		X	X
FTS	Failure to start on demand	Doesn’t start on demand	X		
HIO	High output	Overspeed/output above acceptance	X		
ILP	Internal leakage—process medium	Leakage internally of process fluids			X
ILU	Internal leakage—utility medium	Leakage internally of utility fluids	X		X
INL	Internal leakage	Leakage internally of process or utility fluids	X		
LCP	Leakage in closed position	Leakage through valve exceeding acceptance criteria when closed		X	
LOO	Low output	Delivery/output below acceptance	X		
OHE	Overheating	Machine parts, exhaust, cooling water	X		
OTH	Other	Failure modes not covered above; Specify in comment field	X	X	X
PCL	Premature closure	Spurious closure of valve without command		X	
PDE	Parameter deviation	Monitored parameter exceeding limits, e.g., high/low alarm	X		

**Table 5:** Severity table.

Severity Categories		People	Assets/Operational Continuity	Environment
I	Insignificant	No injuries or, at most, first aid cases	Minor damage to equipment without compromising operational continuity	Insignificant damage
II	Marginal	Minor injuries	Minor damage to systems/equipment	Minor damage
III	Moderate	Serious intramural injuries or minor extramural injuries	Moderate damage to systems	Moderate damage
IV	Critical	Intramural fatality or serious extramural injuries	Severe damage to systems (slow repair)	Severe damage with localized effect
V	Catastrophic	Multiple intramural fatalities or extramural fatality	Catastrophic damage potentially leading to loss of the industrial facility	Severe damage in sensitive areas or spreading to other locations

**Table 6:** Frequency table.

Frequency Category		Frequency Description	Frequency Code
1	Extremely Remote	Possible but with no references in the industry	A
2	Remote	Not expected to occur, although there are references in similar industry facilities	B
3	Unlikely	Unlikely to occur during the service life of a set of similar facilities	C
4	Likely	Possible to occur once during the service life of the facility	D
5	Frequent	Possible to occur multiple times during the service life of the facility	E

**Table 7:** Detection table.

Detection Category RPN-DPR		Designation	Criterion	Interpretation
5	1	Very Low	No resources for detection (example: none of the resources mentioned in categories 1, 2, and 3 are present).	No tag or inspection
4	2	Low	Few resources for detection (example: only perceptible by human senses).	Human inspection (visual, manual, perception)
3	3	Moderate	Availability of inspection resources (example: measuring equipment).	Inspection using measuring equipment
2	4	High	Availability of inspection and monitoring resources (online sensing) for only one parameter.	Monitoring by only one type of tag
1	5	Very High	Availability of inspection and monitoring resources (online sensing) for multiple parameters.	Monitoring by more than one type of tag (e.g., temperature, flow rate, status, and pressure)

### 3.6. Setting Failure Modes

The failure modes used for this case were also derived from ISO 14224, specifically from the failure mode tables B.7—Mechanical equipment, B.8—Electrical equipment, B.10—Subsea equipment, and B.11—Well completion equipment. The primary table used was B.11, as it relates to well completion; the other tables were used to supplement the analysis of components with functions beyond those in B.11. A list of 34 standardized failure modes, with codes from these ISO tables, was established. The following are some of the failure modes used and their adopted definitions. These modes were highlighted due to their potential for different interpretations.

- “STD—structural deficiency”—used for components with a structural function that are subject to extreme effects from pressure, stress, or fluid flow, which may cause abrasion, erosion, cracking, or surface damage.
- “ILP—Internal Leakage—process”—considered the leakage of fluid from a higher external pressure into an internal system with lower pressure.
- “ELP—External Leakage—process” and “ELU—External Leakage—utility”—considered the leakage of fluid from a higher internal pressure into an external system with lower pressure.

Each relationship between component and functional failure may have one or more distinct failure modes, as shown in Table 8. These modes were identified through analysis of failure history, literature, component analysis, and team experience. Functional requirements and expected product performance were also analyzed using the taxonomic glossary and functional diagram. Modes were described for each analysis, enabling a detailed understanding of how the failure occurs.

Failure modes stemming from design errors unlikely to occur under current operational conditions or industry history were discarded. Design-related failures were not the focus of this application. Other unlikely failures were considered, as one objective of the FMMEA is to list all possible failure scenarios.

### 3.7. Setting Failure Mechanisms

ISO 14224 divides mechanisms into categories such as mechanical failures, material failures, instrumentation issues, electrical failures, external influences, and others. In this application, specific rules were developed to standardize interpretations when establishing mechanisms. Since FMMEA often involves large, complex systems with multiple subsystems, experts can easily lose focus. These rules help ensure consistency in the analyses. Key guidelines applied in this case included:

- 2.5 Rupture—Would cause a significant fluid leak in high-pressure hydraulic systems, so this mechanism was not considered for failures related to flow changes.
- 2.3 Erosion—Applied to chemical injection lines, as there is potential for oil contamination (solid particles) over long piping distances.
- 2.2 Corrosion and 2.3 Erosion—Considered for the “STD—structural deficiency” mode, as this association directly relates to the system’s functions and mechanisms (metal surfaces and fluid flow).
- 2.6 Fatigue—Not applicable to chemical injection lines and valves, as these components are not under mechanical stress.
- 2.1 Cavitation—Excluded for components installed submerged or at the well bottom, as high operating pressures prevent cavitation.
- 3.2 Signal Absence—Related to the signal sender and transmitter.
- 1.4 Deformation—Not considered for association with leakage mode, as the components are metal. Deformation may cause obstruction but not internal or external leakage. Exception: for the Penetrator/Connector, this rule does not apply and both external and internal leakage may occur.
- 2.4 Wear—Mechanisms involving metal-to-metal contact, or fluid with solid contaminants under high-pressure flow, with erosion effect, were considered.
- Generic mechanisms (General, Other, Unknown) from ISO 14224 table B2 were avoided as they may hinder the identification of related causes, modes, and events.

The use of identical terminology for both mode and mechanism was also avoided, as this would introduce redundancy without adding value to the analysis.

As illustrated in the example of the ICV with the functional failure “1.4 Entry of undesirable fluids into the well” (Table 8), all failure mechanisms that occurred or were possible for each failure mode were identified. These occurrences were identified from failure history in the project database. Possible mechanisms were identified based on component analysis, structure, operation, and environment. Information was gathered from manuals, manufacturer documentation, and team experience.

Each association (component, functional failure, mode, and mechanism) was initially proposed by at least three experts, discussed in a group of about ten professionals, validated by the group supervisor, and finalized in review meetings. About four proposal meetings were held per subsystem, ten group meetings for feedback and additional contributions, and three review meetings after supervisor validation. The system considered includes five subsystems, 25 components, and 35 functional failures. Table 8 presents only one example.

### 3.8. Setting Failure Causes

The failure causes identified at this stage were derived from the standardized list in ISO 14224, specifically Table B.3, which categorizes failure causes into five groups: (i) design-related, (ii) manufacturing/installation-related, (iii) operation/maintenance-related, (iv) management-related, and (v) other causes.

For each failure mechanism defined previously, one or more failure causes had to be identified and associated. To ensure consistency, experts needed to keep in mind the functional failure and failure mode being analyzed, and to distinguish between the failure cause (the underlying “root” cause) and the failure mechanism (the apparent and observed form). Given that the FMMEA framework is detailed and often extensive, mistakes can easily occur during its development.

Generic failure causes from Table B.3, identified by subdivision codes 1.0, 2.0, 3.0, 4.0, 5.0, 5.1, 5.5, and 5.6, were excluded, as they did not contribute significantly to root cause identification. Less likely failure causes, on the other hand, were included.

Table 8 demonstrates an example of establishing failure causes for the ICV, part of the Flow Control Subsystem, considering the functional failure “entry of undesirable fluids into the well.” During several working meetings, the team reached a consensus on the most suitable failure causes for each mechanism. These causes are shown in the last column. The way the failure sequence information was visualized enabled a logical connection between FMMEA items, which is essential for ensuring consistent associations.

### 3.9. Risk Evaluation and Prioritization

To establish intervention priorities for failures identified in the FMMEA using an indicator (RPN or DPR), severity, frequency, and detectability scales must be defined. For the ICV case study, the RPN and DPR scores were calculated as follows:

- Component: Interval Control Valve;
- Functional failure: 1.4—Entry of undesirable fluids into the well;
- Failure mode: ILP—Internal leakage—process;
- Mechanism: Adhesion.

Each severity, frequency, and detectability category was assigned a brief description, providing more detail on the associated characteristic, the established degree of occurrence, and the mechanism’s inspection or sensing capability.

For the failure sequence mentioned, the following values were assigned: severity 2 (marginal: minor damage to systems/equipment), frequency 5 (possible to occur several times during the installation’s lifetime), and detectability 2 (high detectability). The product of these factors yields an RPN of 20.

To prioritize monitoring capability, the detection scale was inverted. For the development of a DT with predictive capabilities, which requires continuous monitoring, therefore, failure sequences must be detectable or sensor-monitored. However, system constraints prevent installing new sensors.

For the DPR calculation, the values for severity, frequency, and detection were: 2 (marginal: minor damage to systems/equipment), 5 (possible to occur several times during the installation’s lifetime), and 4 (high detectability). Multiplying these indices resulted in a DPR of 40, representing the priority associated with detection capability for the failure sequence. Table 9 shows the results obtained for RPN and DPR, along with their descriptions, for the analyzed failure sequence.

**Figure 8:**
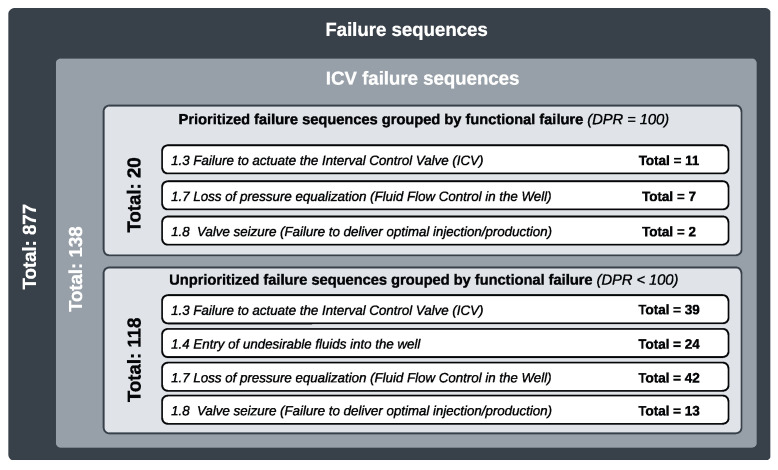
Prioritized and unprioritized failure sequences.

**Figure 9:**
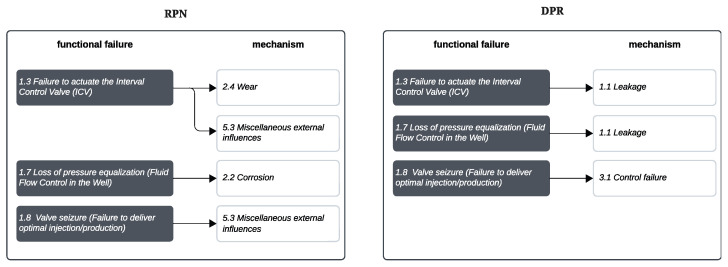
Comparison of failure sequences prioritization approaches.

When analyzing this failure sequence, based on the DPR and RPN results and considering the set of 311 failure trains defined by the project team, it presents a low level of priority. In comparison with sequences that, for example, obtained DPR values above 80, this sequence is classified at level 4 on a scale ranging from 0 to 4, where 0 represents the highest priority. Figure 8 illustrates the priority hierarchy of the sequences based on the DPR. To provide a comparison, Figure 9 demonstrates the difference in the failure sequences prioritization that results from applying RPN versus DPR.

### 3.10. General Review and Validation by Third Parties

The general review was conducted with input from the entire team. Initially, in a working meeting, the team verified the pertinence of the functional failures identified for each component. As the analysis progressed, adjustments (addition or removal of one or more functional failures) were made. The entire process considered the failure sequences stemming from the functional failure, including all associated modes, effects, mechanisms, and causes.

Next, a team expert provided comments for each sequence linked to a functional failure, analyzing each component. These comments focused on the relevance and consistency of the relationships between the associated failure modes, mechanisms, and causes, as well as the accuracy of the descriptions for failure modes and mechanisms in the FMMEA. The feedback included reflections on item adequacy and suggestions for improvement.

Then, team members with more expertise in FMMEA provided further insights into the adequacy of the mode and mechanism descriptions and the consistency of the associations in each identified failure sequence. The feedback emphasized the congruence of failure mechanisms and causes with the failure modes established for each component or functional failure.

Based on the feedback, working meetings were held for team reflection and necessary adjustments to the failure hierarchy. Once the refinements were complete, the document was checked for spelling errors and formatting. At this point, a consolidated and updated version of the entire FMMEA was generated and submitted to external reviewers.

Third-party validation was performed by external members of the team who worked directly with ICSs. Collaboration with the external team enabled evaluation of the pertinence of failure sequences based on field information.

After methodological alignment, internal and external teams validated the FMMEA, following the same verification procedures.

All improvement suggestions made by the external team during working meetings were recorded. Once the analyses by the expert team on the ICS were completed, the final suggested adjustments were made. Upon completion of third-party validation, the FMMEA was finalized and the final version of the failure hierarchy was obtained.

## 4. Final Remarks

Through the presentation of the FMMEA framework in the context of the O&G industry, this study demonstrated how a failure identification approach that considers failure mechanisms can significantly improve the reliability of DTs and their effectiveness in predictive maintenance strategies. In this way, it was possible to achieve the main objective of this research. The FMMEA framework proved effective in providing structured and detailed information on failure modes, mechanisms, and causes, and it can support the integration of relevant data into virtual models, helping to improve the predictive capability and overall usefulness of the DT during the asset’s lifecycle. The case study validated the practical applicability of FMMEA, showing how this framework can be integrated into ICSs to identify failures at early stages or even before they occur, thus mitigating operational risks. Furthermore, the adjustment made to the traditional RPN (Risk Priority Number) by adopting the DPR (Detectable Priority Risk) for risk prioritization in DT applications proved to be timely. In complex systems where sensorization and monitoring cannot be modified, this strategy becomes even more effective. Additionally, the study contributes by offering specific and standardized guidelines, as well as a step-by-step process for applying FMMEA in O&G contexts, which can be adapted to other sectors.

This FMMEA was designed to support DT development, not to create an improvement action plan. It effectively enhances DT predictive capabilities.

This study fills a notable gap in O&G failure identification, particularly for DT development. FMMEA aids risk management and maintenance optimization, promoting safer, more efficient operations.

Despite the advancements brought by this study, some limitations should be considered. First, the application of failure identification methods in DTs is relatively new and requires further validation in different contexts and systems within the O&G industry, as this study was applied to a system in this sector. Moreover, the dependence on data quality for the effectiveness of FMMEA is a limitation, since incomplete or inaccurate data can compromise the results of the analysis.

For future research, it is suggested to explore the integration of FMMEA with other data analysis techniques, such as machine learning and artificial intelligence, to enhance the accuracy and automation of the failure identification process. Furthermore, it would be interesting to investigate the application of FMMEA as a supporting tool for the creation of DTs in other sectors, considering companies of different sizes.

## Figures and Tables

**Table 8:** Failure sequence with failure causes.

Subsystem	Functional Failure	Associated Component	Failure Mode	Failure Mode Description	Failure Mechanism	Failure Cause
1—Flow Control Subsystem	1.4—Entry of undesirable fluids into the well	Internal Control Valve (ICV)	ILP—Internal leakage— process	Represents the leakage of fluids (oil, gas, condensate, water) to different production zones	1.3—Clearance/ alignment failure	2.1—Fabrication Failure
						2.2—Installation Failure
						3.4—Expected wear and tear
					1.5—Looseness	2.2—Installation Failure
						3.4—Expected wear and tear
					1.6—Sticking	2.2—Installation Failure
					2.4—Wear	1.1—Improper capacity
						2.1—Fabrication Failure
						2.2—Installation Failure
						3.4—Expected wear and tear
					2.5—Breakage	1.1—Improper capacity
						2.1—Fabrication Failure
						2.2—Installation Failure
						3.4—Expected wear and tear
			STD—Structural deficiency	Represents material damage (cracks, wear, fractures, corrosion) and reduced physical integrity	1.4—Deformation	1.1—Improper capacity
						2.2—Installation Failure
						3.4—Expected wear and tear
					2.2—Corrosion	1.1—Improper capacity
						2.1—Fabrication Failure
						2.2—Installation Failure
						3.4—Expected wear and tear
					2.3—Erosion	1.1—Improper capacity
						3.4—Expected wear and tear

**Table 9:** RPN and DPR values for the analyzed failure sequence.

Severity Category	Severity Description	Frequency Category	Frequency Description	Detection Category (RPN)	Detection Category (DPR)	Detection Description	RPN	DPR
2	Marginal: Minor damage to systems/equipment	5	Frequent: May occur several times during equipment life	2	4	High: Availability of inspection and monitoring resources (online sensing) for only 1 parameter.	20	40

## Data Availability

The data are contained within the article.

## Abbreviations

The following abbreviations are used in this manuscript:

AIAA	American Institute of Aeronautics and Astronautics
DPR	Detectable Priority Risk
DT	Digital Twin
ETA	Event Tree Analysis
FMEA	Failure Modes and Effects Analysis
FMECA	Failure Mode, Effects, and Criticality Analysis
FMMEA	Failure Modes, Mechanisms, and Effects Analysis
FTA	Fault Tree Analysis
HAZOP	Hazard and Operability Study
ICS	Intelligent Completion System
ICV	Interval Control Valve or Inflow Control Valve
IoT	Internet of Things
ISO	International Organization for Standardization
O&G	Oil and Gas
PHM	Prognostics and Health Management
RPN	Risk Priority Number
